# New Continuous Air Pumping Technique to Improve Clinical Outcomes of Descemet-Stripping Automated Endothelial Keratoplasty in Asian Patients with Previous Ahmed Glaucoma Valve Implantation

**DOI:** 10.1371/journal.pone.0072089

**Published:** 2013-08-16

**Authors:** Chang-Min Liang, Yi-Hao Chen, Da-Wen Lu, Jiann-Torng Chen, Ming-Cheng Tai

**Affiliations:** Department of Ophthalmology, Tri-Service General Hospital, National Defense Medical Center, Taipei, Taiwan; Medical University Graz, Austria

## Abstract

**Background:**

To evaluate the outcomes of Descemet-stripping automated endothelial keratoplasty (DSAEK) with the use of continuous air pumping technique in Asian eyes with previous Ahmed glaucoma valve implantation.

**Methods:**

The DSAEK procedure was modified in that complete air retention of the anterior chamber was maintained for 10 min using continuous air pumping at 30 mm Hg. The primary outcome measurement was graft survival, and postoperative clinical features including, rate of graft detachment, endothelial cell count, intraocular pressure (IOP), surgical time and cup/disc ratio were also recorded.

**Results:**

A total of 13 eyes of 13 patients which underwent modified DSAEK and 6 eyes of 6 patients which underwent conventional DSAEK were included. There was a significant difference in graft survival curves between two groups (*P* = 0.029); the 1-year graft survival rates were estimated as 100% and 66.7% for patients with modified DSAEK and those with traditional DSAEK, respectively. The rate of graft detachment were 0% and 33.3% for the modified DSAEK and conventional DSAEK groups, respectively (*P* = 0.088). The significantly lowered surgical time for air tamponade was noted in the modified DSAEK group compared to that in the conventional DSAEK group [median (IQR): 10.0 (10.0, 10.0) min vs. 24.5 (22.0, 27.0) min; *P*<0.001] Postoperatively, patients in the modified DSAEK group had significantly lower IOP as compared to the conventional DSAEK group [12.0 (11.0, 15.0) mm Hg vs. 16.0 (15.0, 18.0) mm Hg; *P* = 0.047]. Modified DSAEK patients had higher endothelial cell counts as compared to conventional DSAEK patients [2148.0 (1964.0, 2218.0) vs. 1529.0 (713.0, 2014.0)], but the difference did not reach statistical significance (*P* = 0.072).

**Conclusions:**

New continuous air pumping technique in DSAEK can be performed safely and effectively in patients with prior GDDs placement who have corneal failure.

## Introduction

Patients who have had poor glaucoma control with frequent acute attacks and long-term high intraocular pressure (IOP) usually develop irreversible endothelial compromise and chronic corneal edema. [1] Implantation of glaucoma drainage devices (GDDs) have assumed an important role in the surgical treatment of complicated and refractory glaucoma. [2] However, patients with GDDs are at further risk for developing corneal endothelial failure, and corneal transplantation has been the only successful option to preserve vision. [2] Full-thickness penetrating keratoplasty (PKP) is commonly performed for visual rehabilitation; however, drawbacks of the procedure include prolonged visual recovery, risk of wound dehiscence and infection, development of secondary glaucoma, and high rates of graft failure. [3].

Endothelial keratoplasty has been developed an alternative to PKP for the treatment of corneal endothelial decompensation. [4] Recently, Descemet’s stripping automated endothelial keratoplasty (DSAEK) has been developed, and is rapidly becoming the procedure of choice in patients with corneal disease. [3] Advantages of DSAEK over PKP include rapid healing and visual recovery, smaller incision and hence a stronger wound after healing, better visual acuity, and lower risk of graft failure. [3], [5], [6] Though a relatively new procedure, excellent long-term outcomes have been reported. [7]–[9] Letko et al. [10] reviewed a consecutive series of 1050 primary DSAEK procedures and reported that in cases of unacceptable visual acuity after primary DSAEK repeat endothelial keratoplasty can improve vision in select patients. In addition, good outcomes have been reported with the use of corneal donor tissue that is not suitable for PKP, thus increasing the potential donor pool. [11].

Despite the advantages of DSAEK, the procedure is more challenging in patients with prior glaucoma surgery, and in particular in those with tube shunt placement. [3], [12]–[14] Potential complications in patients with prior tube shunts include the tube interfering with the placement of the graft, the tube providing a potential route for air to escape, inadequate air tamponade, possible graft detachment and papillary block glaucoma, and air migrating behind the pupil. [12].

To overcome the challenges of DSAEK, we developed a modified DSAEK using a continuous air pumping technique. The purpose of this study is to report the clinical outcomes of the modified DSAEK in Asian patients with glaucoma and prior Ahmed tube placement using anterior segment optical coherence tomography (AS-OCT) as an assessment tool.

## Materials and Methods

### Patient Selection

The study was approved by the institutional review board and Independent Ethics Committee of Tri-Service General Hospital, and all patients gave written informed consent for the surgical procedures. As the study was a review of medical records, the requirement of informed consent for the study was waived.

The records of patients with refractory glaucoma and an existing Ahmed glaucoma valve whose IOP was controlled and had a cup/disc ratio<0.7, but required corneal transplantation due to chronic corneal decompensation who had undergone DSAEK from May 2010 to December 2011 were retrospectively reviewed. Patients with preexisting ocular comorbidities that could result in less than optimal visual potential (e.g., optic atrophy, blindness) and those without complete follow-up were excluded. Patients were divided into 2 groups for analysis, those that received the modified DSAEK and those that received conventional DSAEK. The procedures and treatments were the same for both groups, except in the modified DSAEK group the continuous air pumping technique was used.

The presence of graft detachment within one day postoperatively, surgical time for air tamponade, as well as visual acuity, intraocular pressure (IOP), and endothelial cell count (ECC) at 1, 3, 6, and 12 months postoperatively were recorded. Modified surgical maneuvers and time used at the time of the procedure were also recorded. Additionally, the corneal grafts and tube positions were imaged in all cases with the Fourier-domain AS-OCT. The AS-OCT device yields 26000 A-scans per second, and has a depth resolution of 5.0 m using an RTVue system (version 3.0, Optovue, Inc.).

The primary outcome measurement was graft survival, and postoperative clinical features were rate of graft detachment, endothelial cell counts, intraocular pressure (IOP), surgical time and cup/disc ratio.

### Modified DSAEK

**Figure 1:**
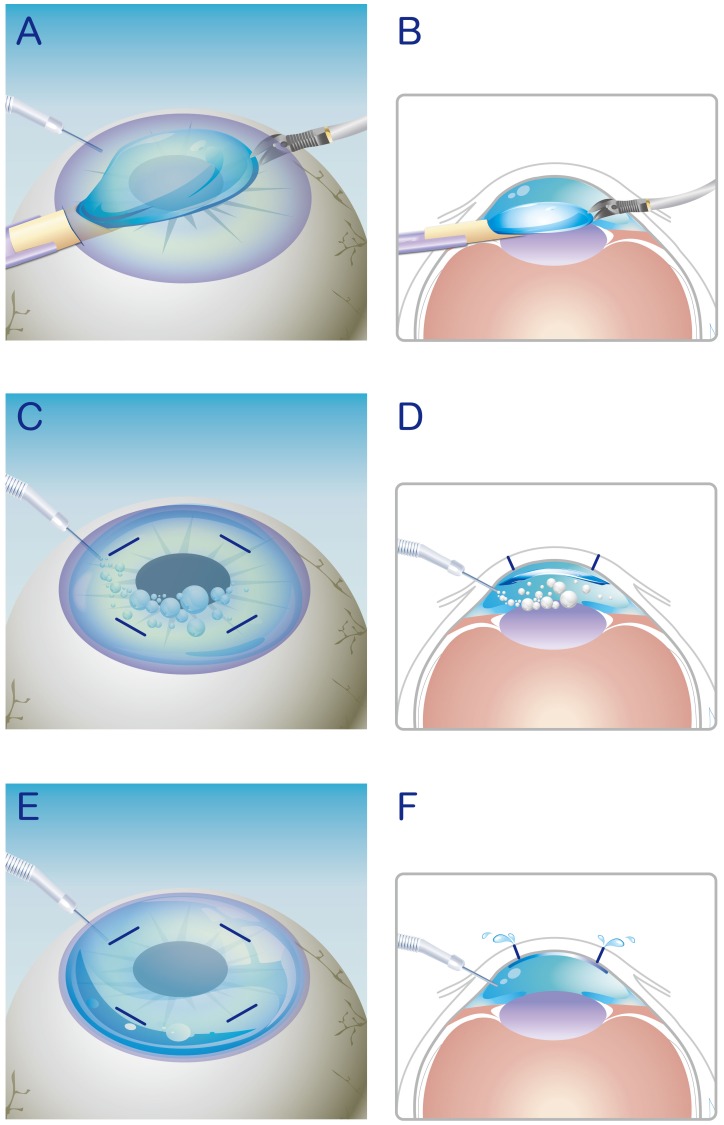
Schematic illustration of the modified DSAEK procedure. A, B) Donor tissue insertion. C, D) Continuous air pumping. E, F) Drainage of interface fluid from venting incision.

**Figure 2:**
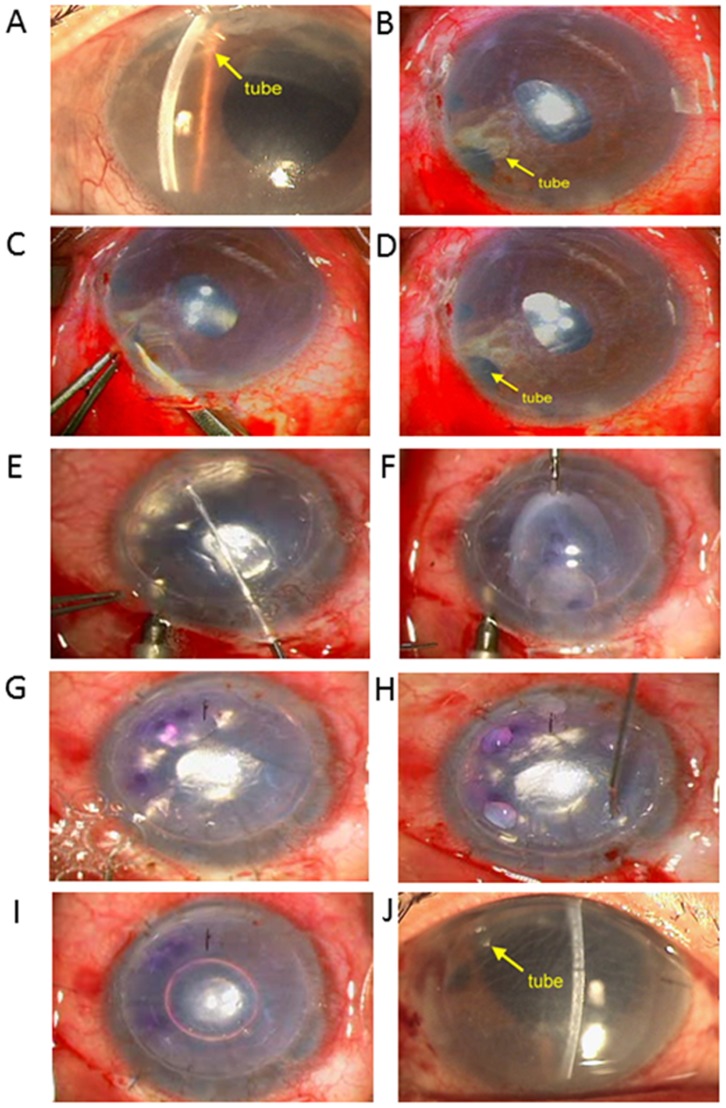
Modified DSAEK procedure. A) Preoperative image. B-D) Tube was cut intraoperatively. E) Descemet’s stripping with 30 mm Hg pressure. F) Donor tissue insertion. G) Continuous air pumping (30 mm Hg for 10 min). H) Drainage of interface fluid from venting incision. I) Residual air bubble left at the end of the surgery. J) Image at 1 week postoperatively.

The modified DSAEK in this study involved continuous air pumping/inflation to enhance graft adherence, rather than the commonly used single air pumping/inflation. Single air pumping has been associated with frequent air migration to the posterior eye and may lead to increased IOP. [3], [12], [13] The surgical technique is illustrated in Figures 1 and 2. DSAEK was performed through a 5-mm tunneled scleral incision by scoring Descemet’s membrane, and stripping it from the posterior corneal stroma. The peripheral recipient stromal bed was subsequently scraped to promote adherence. All cornea buttons were from domestic donors. The donor tissue was trephinated with a 7.0–8.5 mm trephine to obtain a smaller graft to avoid contact with the tube. In cases in which DSAEK was performed after a PKP, the diameter of the donor corneal punch used was the same as the diameter of the PK. The donor cornea button was folded and inserted into the recipient eye using Tan EndoGlide (Coronet, Network Medical Product Ltd., UK), then it was unfolded by filling the anterior chamber with balanced salt solution followed by the injection of an air bubble to complete the unfolding.

Complete air retention of the anterior chamber was maintained for 10 minutes in each study case using continuous air pumping to maintain the IOP at approximately 30 mm Hg as determined by Tonopen (Fig. 2G). Continuous air infusion was given using the ALCON Accurus 400 VS Vitrectomy. Four full-thickness surface stab venting incisions were made peripherally to drain the interface fluid. Ahmed tube revision was performed when necessary. The Ahmed tubes in the anterior chamber of 7 cases were revised in order to provide enough space for the graft and prevent trauma to the endothelium. The tube was cut in 4 cases in which the tip did not extend 1–2 mm beyond the cornea. The tube in the 3 case was removed from the anterior chamber, shorted, and re-inserted in the sulcus (Fig. 2B–E). Synechiolysis was performed if peripheral anterior synchiae (PAS) were visible. The scleral incision was closed with 4–5 interrupted sutures. The IOP was checked by Tonopen in all cases, and it was ensured that the residual air bubble left at the end of the surgery was not attached to the papillary margin to avoid papillary block glaucoma. The patients were kept in a supine position and observed in the recovery room for 1 hour. Preoperative position of the Ahmed valve and postoperative position of the graft and Ahmed valve were assessed with AS-OCT.

### Statistical Analysis

Continuous variables were summarized by medians and interquartile ranges (IQR; 25^th^ and 75^th^ percentiles) for small sample size and non-normally distributed data; categorical variables were expressed by counts and percentages. The primary outcome was defined as graft survival estimated by Kaplan-Meier method, and compared by the log-rank test. The secondary outcomes were defined as postoperative clinical features, such as rate of graft detachment, surgical time for air tamponade, IOP, and endothelial cell count. The difference of IOP was calculated as postoperative IOP minus preoperative IOP. The comparisons between patients with modified DSAEK and those with conventional DSAEK were performed by the Mann-Whitney U test for continuous variables, and by the Fisher’s exact test for categorical variables. The statistical analyses were performed with SAS software version 9.2 (SAS Institute Inc., Cary, NC). A two-tailed *P*<0.05 indicated statistical significance.

## Results

A total of 13 eyes of 13 patients which underwent modified DSAEK and 6 eyes of 6 patients which underwent conventional DSAEK were identified and included in the analysis. All eyes had prior Ahmed valve tube shunt placement. The median age of the patients who received modified DSAEK was 66 years (IQR, 57 to 68 years) and that of the patients who received conventional DSAEK was 65.5 years (IQR, 57 to 69 years). The clinical features of the 2 groups were not different (Table 1).

**Table 1:** Characteristics of patients who received modified DSAEK and those who received conventional DSAEK.

	Modified DSAEK (*n* = 13)	Conventional DSAEK (*n* = 6)	*P*-value
**Age (y)** a	66.0 (57.0, 68.0)	65.5 (57.0, 69.0)	0.930†
**Gender** b			
Female	6 (46.2)	4 (66.7)	0.629‡
Male	7 (53.9)	2 (33.3)	
**OD/OS** b			
OD	8 (61.5)	3 (50.0)	1.000‡
OS	5 (38.5)	3 (50.0)	
**Type of glaucoma** b			
Open angle	4 (30.8)	0 (0.0)	0.292‡
Uveitic	4 (30.8)	1 (16.7)	
Closed angle	5 (38.5)	5 (83.3)	
**Prior PK** b			
0	4 (30.8)	0 (0.0)	0.119‡
1	7 (53.9)	3 (50.0)	
2	1 (7.7)	3 (50.0)	
3	1 (7.7)	0 (0.0)	
**Ahmed tube location** b			
Anterior Chamber	10 (76.9)	5 (83.3)	1.000‡
Posterior chamber	3 (23.1)	1 (16.7)	
**Follow-up time (month)** a	15.0 (10.0, 15.0)	18.5 (4.0, 19.0)	0.427†

a Continuous data are presented as median (IQR),

b categorical data are presented as number (%).

† Mann-Whitney U test;

‡ Fisher’s exact test.

DSAEK, Descemet’s stripping automated endothelial keratoplasty.

**Figure 3:**
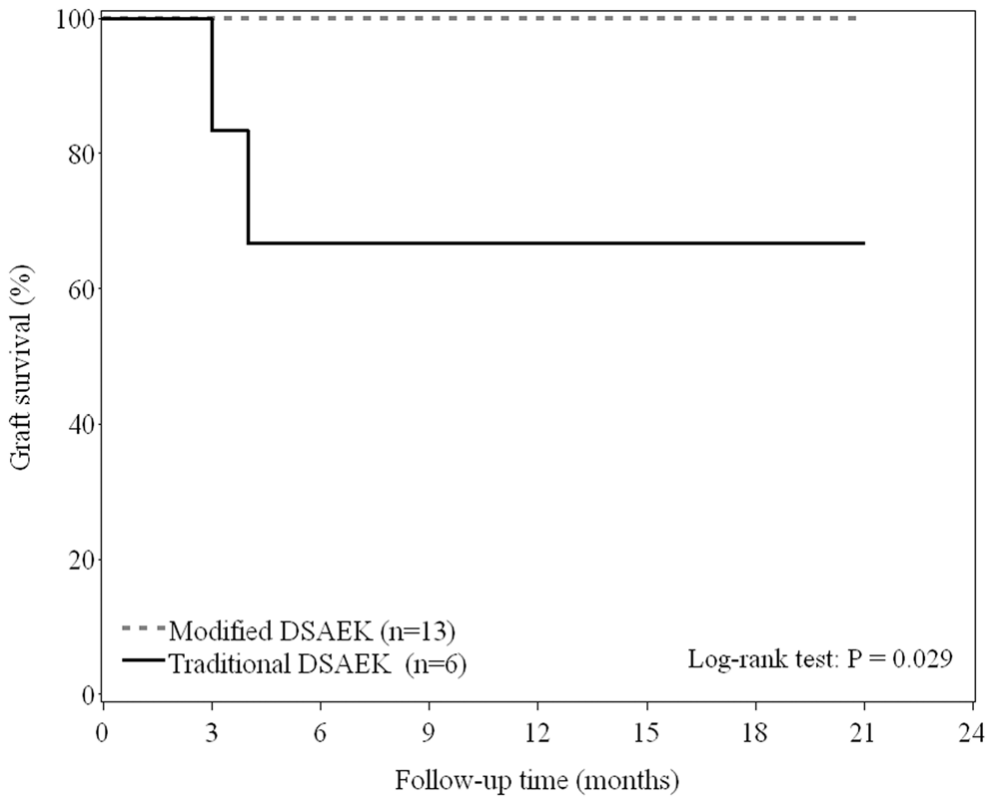
Estimated graft survival determined by Kaplan-Meier method.

Kaplan-Meier survival curves of graft survival are shown in Figure 3. There was a significant difference in graft survival curves between two groups (*P* = 0.029); the 1-year graft survival rates were estimated as 100% and 66.7% for patients with modified DSAEK and those with traditional DSAEK, respectively. Postoperative clinical features results are shown in Table 2. In study group, all the grafts were attached on postoperative day 1 and corneal edema was resolved. In control group, two patients that experienced graft detachment underwent rebubbling with C3F8 on postoperative day 2 resulting in attachment of graft. The rate of graft detachment were 0% and 33.3% for the modified DSAEK and conventional DSAEK groups, respectively (P = 0.088). There were no cases of IOP elevation or papillary block immediately after surgery, but in 1 patient in control group loss of pressure control developed after the DSAEK. This patient required repeat Ahmed valve implantation to regain normal pressure control. No patients in the study developed swelling of the perioribital area.

**Table 2:** Postoperative clinical features compared between patients who received modified DSAEK and those who received conventional DSAEK.

Secondary outcomes	Modified DSAEK (*n* = 13)	Conventional DSAEK (*n* = 6)	*P*-value
Rate of graft detachment^a^	0/13 (0%)	2/6 (33.3%)	0.088
Preoperative IOP (mm Hg)^b^	14.0 (13.0, 18.0)	17.0 (15.0, 18.0)	0.627†
Postoperative IOP (mm Hg)^b^	12.0 (11.0, 15.0)	16.0 (15.0, 18.0)	0.047†
Difference of IOP (mm Hg)b,c	−2.0 (−3.0, −1.0)	0.0 (−2.0, 2.0)	0.289†
Endothelial cell count^b^	2148.0 (1964.0, 2218.0)	1529.0 (713.0, 2014.0)	0.072†
Surgical time for air tamponade^b^	10.0 (10.0, 10.0)	24.5 (22.0, 27.0)	<0.001†

a Categorical data are presented by counts and percentage.

b Continuous data are presented as median (IQR).

c Differences are calculated as postoperative IOP minus preoperative IOP.

† Mann-Whitney U test.

DSAEK, Descemet’s stripping automated endothelial keratoplasty; IOP, intraocular pressure.

After receiving DSAEK, patients in the modified DSAEK group had significantly lower IOP as compared to the conventional DSAEK group [12.0 (11.0, 15.0) mm Hg vs. 16.0 (15.0, 18.0) mm Hg; *P* = 0.047]. Moreover, patients in the modified DSAEK group had higher endothelial cell counts as compared to the conventional DSAEK group [2148.0 (1964.0, 2218.0) vs. 1529.0 (713.0, 2014.0)], but the difference did not reach statistical significance (*P* = 0.072). Surgical time for air tamponade showed significant decrease in the modified DSAEK group compared to that in the conventional DSAEK group [median (IQR): 10.0 (10.0, 10.0) min vs. 24.5 (22.0, 27.0) min; *P*<0.001]. Other secondary outcome measures were not different between the groups (Table 2).

**Figure 4:**
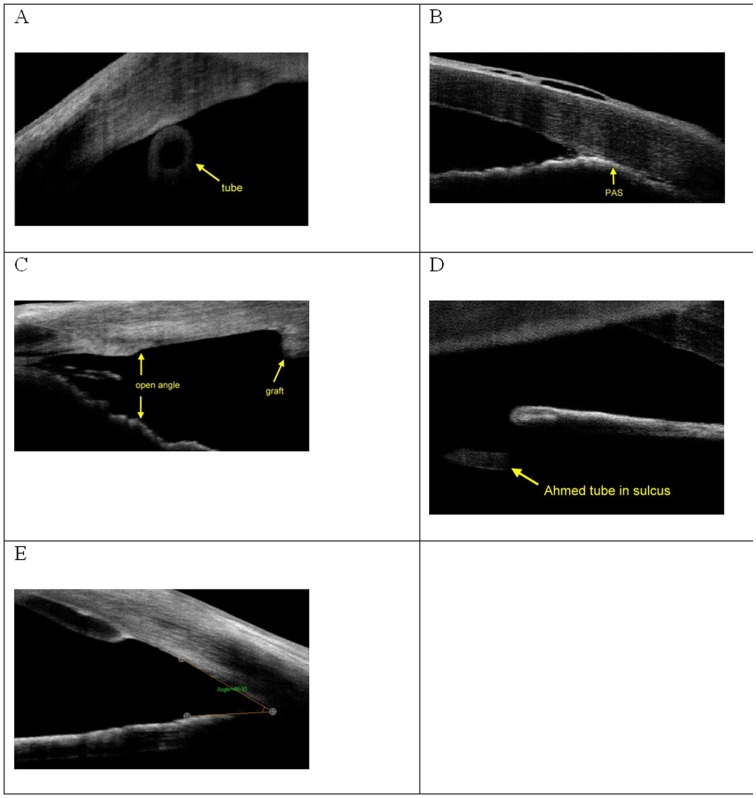
Postoperative follow-up AS-OCT images. A) Ahmed tube position close to the endothelium causing endothelial failure. B) Peripheral anterior synechiae (PAS). C) Wide open angle after synechiolysis and air tamponade. D) Position of Ahmed tube. E) Position of graft and evaluation of angle.

Preoperative position of the Ahmed valve and postoperative position of the graft and Ahmed valve were assessed with AS-OCT for all patients. Preoperative AS-OCT demonstrated the Ahmed tube position close to the endothelium causing the endothelial failure (Fig. 4A). Postoperative AS-OCT revealed the wide open angle after synechiolysis and air tamponade (Fig 4B, C). The trimmed Ahmed tube was also visible with good function in AS-OCT image (Fig. 4D). Additionally, AS-OCT also allowed easily evaluating the angle (Fig. 4E).

## Discussion

Patients with refractory or intractable glaucoma may receive implantation of a glaucoma drainage device; however, endothelial failure and chronic corneal edema may still develop in these patients requiring subsequent corneal transplantation as the ultimate treatment for preserving vision. In this study we have reported good outcomes in 13 patients with prior Ahmed valve placement who underwent a modified DSAEK with the use of a continuous air pumping technique. In our series, there was no primary graft failure, no detachment of graft, no postoperative increase of IOP, no papillary block glaucoma, and no postoperative increase of cup/disc ratio.

DSAEK has rapidly gained acceptance as the preferred procedure in patients with corneal disease. However, the most common postoperative complication after DSAEK is donor detachment and this has been shown to depend on the surgeon’s experience and complexity of the patient, with higher rates at the initial portion of the learning curve. [3] In a recent report with a larger study population, Goshe et al. [15] found the rate of graft dislocation in patients with prior glaucoma surgery was significantly higher than in control eyes (9% vs. 2%; *P = *0.008). This complication eventually increased the failure rate of graft.

DSAEK, however, can be challenging in patients who have had prior tube shunt procedures and potential problems include the tube interfering with the placement of the graft, the tube providing a potential route for the air to escape, inadequate air tamponade, possible graft detachment and papillary block glaucoma, and air migrating behind the pupil. [12].

Despite these potential challenges, a number of recent reports have shown good outcomes of DSAEK in patients with prior placement of GDDs. [13], [14], [16]–[18] Kim et al. [13] performed DSAEK in 11 eyes with GDDs and reported that though the procedure was more challenging, the presence of GDDs was not a contraindication to DSAEK. In one of the larger series, Phillips et al. [14] performed DSAEK in 19 eyes with prior trabeculectomies and 9 with GDDs and compared the results with 431 eyes which had DSAEK without prior surgeries. Though the intraoperative complication rate in the study group (7.1%) was higher than in the control group, there were no primary graft failures in the study group and the dislocation and graft decentration rates were both 3.6%. Our limited result showed even lower complication rate. Quek et al. [16] retrospectively analyzed 47 eyes with pre-existing glaucoma which underwent DSAEK and reported that IOP in glaucomatous eyes undergoing DSAEK can be controlled with minimal increase after DSAEK and eyes with previous filtration surgery required fewer medications to control elevated IOP than eyes that had not had previous surgery. Esquenazi et al. [17] and Riaz et al. [18] each reported 1 patient with prior Ahmed glaucoma valve implantation who underwent DSAEK successfully with good IOP control and graft survival. Other authors have reported successful DSAEK after failed PKP, [19] and good outcomes of trabeculectomy after DSAEK. [20] Anshu et al. [21] reviewed 835 cases of DSEK and found that patients with medically managed glaucoma had better 5-year graft survival than those with surgically managed glaucoma. Interestingly, in another study by Anshu et al. [22] the authors reported that patients who received Descemet’s membrane endothelial keratoplasty (DMEK) had a significantly reduce risk of transplant rejection than those who received DSEK or PK.

The continuous air pumping technique is primarily for tamponading the graft and maintaining the anterior chamber deep, because these are 2 challenges for the conventional air injection technique in DSAEK. In conventional DSAEK, air leakage will result in insufficient IOP which can result in poor graft attachment and drainage of the interface fluid, while air migrating behind the pupil with uncontrolled single air pumping can result in excessively high IOP which can lead to retinal ganglion cell damage and compromise of the retinal circulation. Our continuous air pumping technique can avoid these 2 problems. Additionally, it can also lessen the operative time spent in air tamponade. We used 10 minutes to maintain graft attachment, which is less than the previously reported 30 to 60 minutes [23], [24] spent for addressing intraoperative complications such as shallow anterior chamber and high IOP. Prolonged surgical time, repeated entrance of instrument, and shallow anterior chamber may cause more mechanical endothelial damage resulting in graft failure. Our results also showed that the postoperative cup/disc ratio did not significantly change. We believe that the continuous air pumping technique could be performed safely in glaucoma patients.

In this study, the post-operative IOP was maintained at therapeutic levels in all patients except 1, and 3 patients had a decreased requirement of glaucoma medications after surgery. The possible mechanisms for the control of IOP and graft success included the Ahmed tube provides effective IOP control postoperatively and synechiolysis and intracameral air tamponade during DSAEK not only supports the donor tissue, but also expands the anterior chamber angle. This wide open angle may induce functional recovery of the trabecular meshwork and improve aqueous humor drainage.

AS-OCT is a newly developed method for measuring the anterior chamber angle that is safe and simpler to perform than gonioscopy. We recently reported that AS-OCT provided excellent reproducibility for the quantitative measurement of anterior chamber angles before and after cataract surgery. [25] In this study, we used AS-OCT to assess the variation in angle changes related to DSAEK and found that the technique is useful to determine tube position when there is corneal opacity near the limbus.

Limitations of this study include a small number of patients, relatively short follow-up period, and all surgeries were performed at a single center.

### Conclusions

The results of this study indicate that modified DSAEK can be performed safely and effectively in patients with glaucoma and prior GDDs placement who have corneal failure. The procedure has a low rate of complications and results in good IOP control.
